# Vitamin D Supplementation and COVID-19 Outcomes: Mounting Evidence and Fewer Doubts

**DOI:** 10.3390/nu14173584

**Published:** 2022-08-31

**Authors:** Arrigo F. G. Cicero, Federica Fogacci, Claudio Borghi

**Affiliations:** 1 IRCCS AziendaOspedaliero-Universitaria di Bologna, 40138 Bologna, Italy; 2 Medical and Surgical Sciences Department, Alma Mater Studiorum University of Bologna, 40100 Bologna, Italy; 3 Hypertension and Cardiovascular Risk Research Group, Medical and Surgical Sciences Department, Sant’Orsola-Malpighi University Hospital, 40138 Bologna, Italy

**Keywords:** Cholecalciferol, COVID-19, immune function, mortality, SARS-CoV-2

## Abstract

The coronavirus disease 2019 (COVID-19) has already killed more than 6 million people around the world. A growing body of epidemiological evidence suggests that low 25-hydroxy vitamin D (25-OH-vitamin D) plasma levels are associated with an increased risk of developing COVID-19 and —most importantly—with a higher risk of developing more severe COVID-19 and dying. On the other hand, vitamin D supplementation during the early phases of COVID-19 has been related to a decreased length of hospital stay, less frequent need for oxygen, and a reduced mortality rate in inpatients. This seems to be particularly true when high dosages are used. In light of this evidence, further studies are needed to define the best timing for vitamin D supplementation and the most effective dosage schedule.

## 1. Introduction

Severe acute respiratory syndrome coronavirus 2 (SARS-CoV-2) is a highly transmissible and virulent human-infecting coronavirus that emerged in late 2019 and that causes a (mainly) respiratory disease called coronavirus disease 2019 (COVID-19), which has presumably killed more than 6 million people around the world, to date. Effective vaccines and antiviral drugs are now available for the prevention and treatment of COVID-19, which had previously massively impacted global public health [1] and caused widespread disruption to daily life [2]. During the acute phase of the pandemic, a number of dietary supplements were tested or suggested to promote immune defenses against SARS-CoV-2, even though most of them were not supported by strong clinical evidence of efficacy [3].

In particular, since the onset of the SARS-CoV-2 pandemic in late 2019, an impressive increased use of vitamin D supplements has been observed in some countries [4]. Beyond the emotional reaction of the market, this dietary supplement is probably the only one supported by a considerable amount of efficacy and by any safety data reported forhumans.

## 2. Vitamin D Levels and COVID-19 Prognosis

In urban areas, vitamin D deficiency has been linked to an increased risk of both COVID-19 incidence and related mortality [5].

A meta-analysis of 23 studies, including 2692 SARS-CoV-2 patients with a mean age of 60.8±15.9 years, has shown that plasma 25-hydroxy vitamin D (25-OH-vitamin D) deficiency is associated with an increased risk of developing severe SARS-CoV-2 disease (relative risk (RR): 2.00; 95% confidence interval (CI): 1.47, 2.71) and mortality (RR: 2.45; 95% CI: 1.24, 4.84) [6]. In a larger meta-analysis, including 20 studies and 12806 patients, the RR for mortality, intensive care unit (ICU) admission, ventilator support, and hospital stay length have been found to be, respectively, 1.49 (95%CI: 1.34, 1.65), 0.87 (95%CI: 0.67, 1.14), 1.29 (95%CI: 0.79, 1.84), and 0.84 (95%CI: −0.45, 2.13) [7]. Other studies conducted after these meta-analyses have confirmed that low plasma 25-OH-vitamin Disassociated with increased mortality in different settings [8,9], even in the short term [10]. Furthermore, low plasma levels of 25-OH-vitamin D has been associated with more severe COVID-19 even in children [11].

## 3. Observational and Randomized Clinical Trials Supporting the Use of Vitamin D Dietary Supplementation during COVID-19

After pooling the data from 38 studies and 205,565 patients whose information on 25-OH-vitamin D status was available—including 2022 individuals undergoing vitamin D dietary supplementation, with 1197 patients admitted to the ICU (or those who needed invasive mechanical ventilation or intubation and hospital stay) and 910 deaths for COVID-19, a recent systematic review and meta-analysis has shown that dietary supplementation with vitamin D is associated with a significant lower risk of both COVID-19 severe disease (standardized RR (SRR): 0.38, 95%CI: 0.20, 0.72) and mortality (SRR: 0.35, 95%CI: 0.17, 0.70) [12]. Even though a dose–response curve was not included in this meta-analysis and the authors did not suggest using high doses of vitamin D, more recently, other studies have been released, supporting the use of high vitamin D doses in improving the prognosis of patients with COVID-19. In this regard, the interesting article published in *Nutrients* by Ling et al. has reported solid observational data obtained in the setting of three UK (United Kingdom) hospitals. Ling et al. studied a cohort of 986 patients with COVID-19, 151 (16%) of whom had received a vitamin D booster (approximately ≥ 280,000 IU in a period of up to 7 weeks). In the primary cohort of 444 patients, treatment with high-dose vitamin D was associated with a reduced risk of COVID-19 mortality (odd ratio (OR): 0.13, 95%CI: 0.05, 0.35; *p*< 0.001). This observation has been confirmed in a validation cohort of another541 patients with COVID-19 (OR: 0.38, 95%CI: 0.17, 0.84; *p*= 0.018) [13]. Further double-blind randomized clinical trials have demonstrated that lower (but even high) vitamin D doses were associated with significant decreases in hospitalization length and average duration of supplemental oxygen. In particular, Cervero et al. demonstrated the superiority of dietary supplementation with 10.000 IU/day versus 2.000 IU/day vitamin D during a 14-day period to reduce the duration and severity of COVID-19 in a sample of individuals who were hospitalized (N. 85) [14]. In addition, De Niet et al. found that 25.000 IU vitamin D per day over 4 consecutive days followed by 25.000 IU vitamin D per week up to 6 weeks were superior to the placebo in reducing the duration of supplemental oxygen among patients who needed it (*p*= 0.012) and in improving patients’ clinical recovery (*p*= 0.0048) [15].

The COVID-19and Vitamin D TRIAL (COVIT-TRIAL)—a multicenter, randomized, controlled, open-label, superiority trial involving nine medical centers in France and 254 patients with COVID-19—tested the hypothesis that a single high dose of vitamin D (400 000 UI) could exert different effects from a single dose of50.000 UI vitamin D. The main inclusion criteria for this study were age ≥65 years, early SARS-CoV-2 infection(defined as an infection of less than 3 days), and having at least one worsening risk factor for COVID-19 (among age ≥75 years, peripheral capillary oxygen saturation (SpO_2_) ≤94%, or pressure of arterial oxygen to fractional inspired oxygen concentration ratio (PaO_2_/FiO_2_) ≤300 mm Hg). The study showed that the a high dose of vitamin D improved mortality versus a standard dose at day 14 but not at day 28, being also associated with an increased risk of non-serious adverse events [16]. However, in the large COVID-VIT-D multicenter international trial, a single 100.000 UI vitamin D dose at hospital admission improved clinical outcomes only in patients with higher calcifediol plasma levels at the baseline [17].

## 4. Discussion

Vitamin D supplementation could exert the previously discussed positive effects on COVID-19 clinical outcomes by enhancing the innate antiviral immune response and by facilitating the induction of antimicrobial peptides/autophagy, with a critical modulatory role in the subsequent host reactive hyperinflammatory phase during COVID-19. In particular, vitamin D could reduce the cytokine/chemokine storm, regulate the renin–angiotensin–bradykinin system, modulate neutrophil activity, and maintain the integrity of the pulmonary epithelial barrier through the stimulation of epithelial repair and by directly and indirectly decreasing the increased coagulability and prothrombotic tendency associated with severe COVID-19 and its complications [18]. Furthermore, some data suggest that individuals undergoing vitamin D dietary supplementation before SARS-CoV-2 infection were less susceptible to severe disease during infection [19]. Finally, vitamin D supplementation could also balance some negative effects of COVID-19, such as reduced sun exposition, glucocorticoid therapy, and reduced mobility, which are all well-known risk factors for osteoporosis [20].

The preventive effect of vitamin D on respiratory infections is not new. In 2017, the *British Medical Journal* had already published a meta-analysis of 25 randomized controlled clinical trials, including individual data from 10,933 participants showing that vitamin D supplementation significantly decreases the risk of acute respiratory tract infection (OR: 0.88, 95%CI: 0.81, 0.96; *p* < 0.001) [21].

The most recent findings from the VITAL study suggested that vitamin D supplements are unnecessary, extending the observed findings on the risk of bone fracture found in a young population sample without available information on vitamin D status to the whole population [22]. However, in the context of the COVID-19 pandemic, vitamin D supplementation seems to be an effective preventive and therapeutic tool to be considered, in particular for individuals at high-risk of vitamin D deficiency. Furthermore, some authors also suggest a possible role of vitamin D dietary supplementation in the prevention of and care for the so-called “Long COVID”, for which an evidence-based therapy is not yet available [23].

In conclusion, there is a large and rapidly growing number of clinical studies supporting the evidence that vitamin D supplementation could improve the prognosis of patients affected by COVID-19. Since vitamin D dietary supplementation is inexpensive and overall safe, its use should be considered in the majority of patients, even at intermediate-to-high doses.
